# Reaching Pulp Canals

**Published:** 1874-04

**Authors:** 


*Reaching Pulp Canals.*—At a late meeting of the New York Odontological Society, the proceedings of which are published in the *Dental Miscellany*, Dr. Edward Maynard, in answer to a question "how the pulp canal of an inferior molar could best be reached when the cavity of decay was in the posterior approximal surface," advised "filling the cavity of decay as a simple cavity, and then drilling through the grinding surface, or downward and inward through the buccal surface, commencing near the grinding surface, into the pulp cavity, thus sacrificing but a small amount of tooth substance, and not lessening in any degree the strength of the crown."

"Dr. Maynard illustrated his method by diagrams on the black-board.

He related a case in which, several years ago, he had amputated and extracted the posterior half of the crown, and the posterior root of an inferior molar, shaping the crown remaining as a bicuspid—the removed portion of the crown being much decayed, and the extracted root having a persistent abscess. The result was a perfect success, the tooth doing good service for many years.

Also a case of amputating a portion of the root of an inferior incisor, which protruded through the gum. The gum healed over nicely, and three years after the case was doing well. He performed the operation by drilling a small hole *through* the root at the proposed line of amputation ; enlarged it ; then with a hoe-shaped instrumeut cut through the remaining portion at each side of the hole, drawing the shaving of root outwards at each cut."

